# Modified R-CODOX-M/IVAC chemotherapy regimens in Chinese patients with untreated sporadic Burkitt lymphoma

**DOI:** 10.20892/j.issn.2095-3941.2020.0314

**Published:** 2021-08-15

**Authors:** Meiting Chen, Zhao Wang, Xiaojie Fang, Yuyi Yao, Quanguang Ren, Zegeng Chen, Ying Tian, Fei Pan, Xiaoqian Li, Zhiming Li, Qingqing Cai, He Huang, Tongyu Lin

**Affiliations:** 1 Department of Medical Oncology, Sun Yat-sen University Cancer Center, State Key Laboratory of Oncology in South China, Collaborative Innovation Center for Cancer Medicine, Guangzhou 510060, China; 2 Senior Ward/Phase I Clinical Trial Ward, Sichuan Cancer Hospital & Institute, Sichuan Cancer Center, School of Medicine, University of Electronic Science and Technology of China, Chengdu 610041, China

**Keywords:** Sporadic Burkitt lymphoma, modified chemotherapy, toxicity, R-CODOX-M/IVAC, adults

## Abstract

**Objective:**

To characterize modified R-CODOX-M/IVAC-based chemotherapy to lower the severe adverse events in Chinese adult patients with sporadic Burkitt lymphoma.

**Methods:**

We enrolled a retrospective cohort including 123 adult patients with untreated sporadic Burkitt lymphoma from August 2008 to September 2019 at Sun Yat-sen University Cancer Center. We studied a dose-modified and long-course R-CODOX-M/IVAC regimen utilizing a low dose of 1.0 g/m^2^/cycle cyclophosphamide, 2 g/m^2^/cycle methotrexate, 4,500 mg/m^2^/cycle ifosfamide, and 4.0 g/m^2^/cycle cytarabine. Forty-nine patients with low risk disease underwent 4–6 cycles of dose-modified R-CODOX-M-based chemotherapy. Seventy-four patients with high risk disease underwent 6–8 cycles of dose-modified alternating R-CODOX-M/IVAC regimens.

**Results:**

The objective remission was 87.0%. The event-free survival rate and overall survival at 3 years were 81.2% and 92.1%, respectively. Major grade 3–4 adverse events included leukopenia (91.9%), anemia (58.5%), thrombocytopenia (73.2%), and febrile neutropenia (48.8%). A total of 26.0% and 37.4% of patients received red blood cell and platelet transfusions, respectively. We observed 4 cases (3.3%) of septic shock after chemotherapy. Two treatment-related deaths occurred from severe infection.

**Conclusions:**

The modified R-CODOX-M/IVAC chemotherapy regimen was effective for sporadic Burkitt lymphoma in the Chinese population, with a lower toxicity than standard regimens.

## Introduction

Burkitt lymphoma is a highly aggressive rare subtype of non-Hodgkin lymphoma (NHL) with the genetic hallmark of MYC gene translocation. It is recognized as 3 different variants, namely, endemic, sporadic and immunodeficiency associated^1^. Sporadic Burkitt lymphoma, accounting for approximately 1% of adult NHLs, is typically seen in young patients and has a median age at diagnosis of 30 years among adults^2^. During the past 2 decades, survival has improved due to intensive multiagent alternative chemotherapy regimens including cyclophosphamide, vincristine, doxorubicin, high dose methotrexate (CODOX-M), and ifosfamide, and etoposide and high dose cytarabine (IVAC) with central nervous system (CNS) prophylaxis^3^.

However, severe hematological toxicity caused by intensive and short-course chemotherapy occurred in more than 90% of patients. Sepsis occurred in 22% of patients, and the majority of patients required blood product support^2^. Some prospective clinical trials modified the CODOX-M and IVAC regimens, including the omission of day 15 vincristine, a decrease in the dose of methotrexate to 3 g/m^2^, and alternative use of liposomal doxorubicin, although the study included a small sample size^4,5^. Toxicity was slightly reduced in the modified regimens, and the incidence of grade 3/4 neutropenia, anemia, and thrombocytopenia ranged from 60%–72%, while good efficacy and survival were still achieved. The addition of rituximab to the LMBA95 combination chemotherapy protocol, which adopted a lower drug dose and more courses, improved the event-free survival (EFS) and overall survival (OS) of adult Burkitt lymphoma patients without increasing toxicity in a large prospective randomized controlled trial^6^. Some studies tried to reduce the toxicity of chemotherapy by dose modification. Dunleavy et al.^7^ reported similar progression-free survival (PFS) and reduced neutropenia and fever in patients treated with 6–8 cycles of low density infused etoposide, doxorubicin, and cyclophosphamide with vincristine, prednisone, and rituximab (EPOCH-R) compared with patients treated with standard-dose adjusted EPOCH-R. In sub-Sahara Africa, Zuze et al.^8^ showed that modified EPOCH was practical and safe for resource-limited and poor income areas. Similarly, the Cancer and Leukemia Group B study (CALGB) 9,251 and 10,002 modified the dose of cyclophosphamide to 1 g/m^2^/cycle and methotrexate to 1.5 g/m^2^/cycle, resulting in a 4-year EFS of 74% and treatment related mortality of 9%–13%^9,10^. Thus, the modification of the CODOX-M backbone was encouraging.

Real-world data on modified CODOX-M-based chemotherapy among Asian populations with Burkitt lymphoma are limited. Based on previous studies of modified chemotherapy regimens and experience at the Sun Yat-sen University Cancer Center (SYSUCC), we assumed that a prolonged exposure time instead of an increased dose in R-CODOX-M regimens might be suitable for Chinese patients, and would result in reduced toxicity with similar clinical outcomes. Here, we present the results of a retrospective study of modified R-CODOX-M/IVAC regimens in immunocompetent patients with untreated sporadic Burkitt lymphoma.

## Materials and methods

### Patient selection and treatment

From August 2008 to September 2019, we enrolled 123 patients with untreated Burkitt lymphoma at SYSUCC. Eligible patients had histologically confirmed Burkitt lymphoma and had adequate organ function apart from organ function affected by disease. The evaluation included standard laboratory tests, ^18^F-labelled fluorodeoxyglucose positron emission tomography-computed tomography (CT) scans of the whole body, and bone marrow aspiration and biopsy. Their medical records were analyzed for investigation. The study protocol was approved by the ethical committee of SYSUCC (approval number: SZR2019-016). All patients underwent cytological analysis of the cerebrospinal fluid and imaging of the brain. Modified R-CODOX-M/IVAC regimens were administered as described in **Supplementary Table S1**. We mainly reduced the doses of cyclophosphamide (1 g/m^2^/cycle), methotrexate (2 g/m^2^/cycle), ifosfamide (4,500 mg/m^2^/cycle), and cytarabine (4 g/m^2^/cycle). All cycles were repeated at 21-day intervals. Patients were assigned risk according to the definition by Mead et al.^4^. Patients with low risk had all of the following features: (1) normal lactate dehydrogenase (LDH) levels, (2) an ECOG performance status (PS) of 0–1, (3) an Ann Arbor stage of I/II, and (4) no mass more than 10 cm in diameter; all other patients were considered high risk. Low risk patients received 4–6 cycles of R-CODOX-M regimens. High risk patients received 6–8 alternating cycles of R-CODOX-M and IVAC. All patients received CNS prophylaxis with intrathecal dexamethasone, cytarabine, and methotrexate every 3 weeks.

### Toxicity evaluation

Adverse events (AEs) were graded according to the Common Terminology Criteria for Adverse Events, version 4.0. The relative frequency of each AE considered possibly, probably, or likely related to chemotherapy was estimated as the proportion of all toxicity-evaluated cycles in which such toxicities were observed.

### Response assessment

The objective response was sustained for a minimum of 2 consecutive imaging evaluations at least 4 weeks apart. This protocol was developed using the International Working Group Response Criteria for Malignant Lymphoma and revised Cheson Criteria for Response Assessment. Disease was also evaluated using the Lugano Classification^11^. Positron emission tomography-CT was used to assess treatment responses at baseline and after every 2 cycles of chemotherapy. Follow-up CT scans were performed every 6 months for 2 years or until progressive disease (PD).

### Statistical analysis

The study population for all analyses included patients enrolled in the study who had adequate baseline tumor assessments. Descriptive statistics were used to summarize patient characteristics, treatment administration, antitumor activity, and safety, and the results are presented as medians and ranges. Survival was measured from initiation of therapy until death. EFS was measured from the time from treatment initiation to any treatment failure, including disease progression, change in treatment without documented progression, or death. The objective response rate (ORR), PFS, OS, the type, incidence, severity, seriousness, relationship to study medications of AEs, and laboratory abnormalities were also analyzed. A cutoff date of February 20, 2020, was established for analyzing data for this report. EFS, OS, and PFS were assessed using Kaplan-Meier analyses with SPSS statistical software for Windows, version 25.0 (SPSS, Chicago, IL, USA).

## Results

A total of 123 patients were enrolled and treated (**Table 1**). Patients were 18–69 years of age, with 6 patients (4.9%) more than 60 years of age. A total of 49 patients were at low risk, and 74 were at high risk. CNS involvement was confirmed in 2 patients (1.6%) at the time of initial presentation. Eleven patients were identified with bone marrow involvement, and 1 patient was diagnosed with Burkitt leukemia. Among 86 patients who were assessed for MYC, BCL-2, and BCL-6 rearrangements, 75 of them had MYC gene translocations, and MYC gene breaks were detected in 8 cases, while 3 cases were negative for a MYC gene break or rearrangement. Epstein-Barr virus (EBV) was detected using the EBV-encoded small nuclear early region (EBER) *in situ* hybridization in 70 patients, and 24 patients (34%) were EBER positive.

**Table 1:** Characteristics of patients

Characteristics	*n* (%)
Male	80 (65.0%)
Age (years)	
Median (range)	36 (18~69)
Ann Arbor stage at diagnosis	
I	33 (26.8%)
II	30 (24.4%)
III	6 (4.9%)
IV	54 (43.9%)
IPI score	
0~1	61 (49.6%)
2~3	51 (41.5%)
4~5	11 (8.9%)
B symptoms	
Present	25 (20.3%)
Absent	98 (79.7%)
Serum LDH>ULN	50 (40.7%)
Extra-nodal site	
Bowel	32 (26.0%)
Stomach	22 (17.9%)
Bone marrow	11 (8.9%)
Central nervous system	2 (1.6%)
Risk stratification	
Low	49 (39.8%)
High	74 (60.2%)

ULN, upper limit of normal; IPI, international prognosis index; LDH, lactate dehydrogenase.

**Figure 1:**
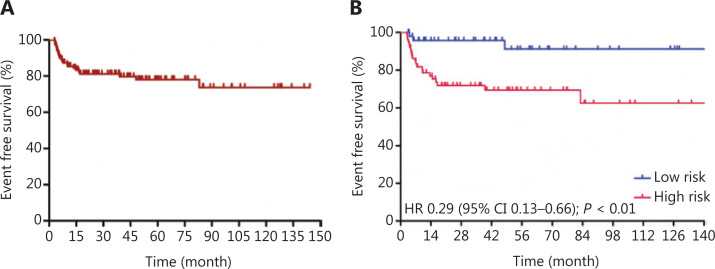
Event-free survival curves for Burkitt lymphoma treated with schedule modified R-CODOX-M/IVAC regimens among total patients (A) and in the low risk and high risk groups (B).

**Figure 2:**
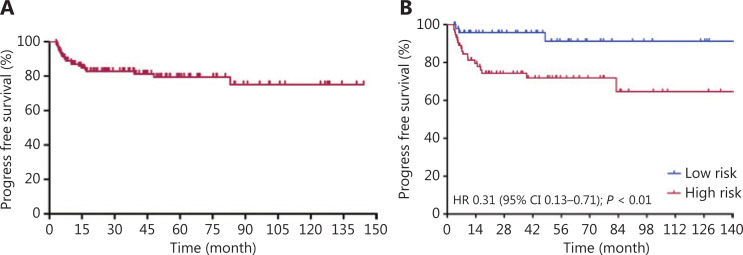
Progress free survival curves for Burkitt lymphoma patients treated with schedule modified R-CODOX-M/IVAC regimens among total patients (A) and in low risk and high risk groups (B).

**Figure 3:**
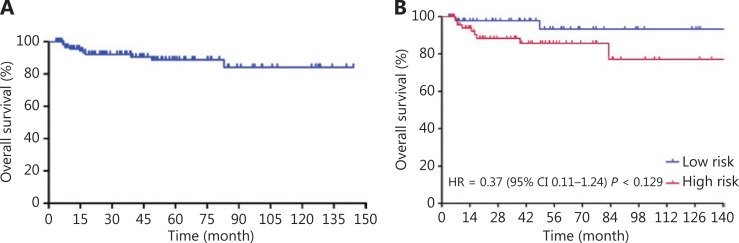
Overall survival curves for Burkitt lymphoma patients treated with schedule modified R-CODOX-M/IVAC regimens among total patients (A) and among low risk and high risk groups (B).

Patients received a median of 6 cycles (range: 4~8) of R-CODOX-M-based regimens. All patients received methotrexate and cytarabine intrathecal therapy except 1 patient who discontinued treatment for lumbar lesions. Thirty patients received 1 cycle of CHOP-like chemotherapy before R-CODOX-M regimens. A total of 111 (90.2%) received rituximab. Twelve patients refused the rituximab combination therapy due to financial considerations. The ORR among all treated patients was 87.0%. The ORRs of the low risk and high risk groups were 98.0% and 89.2% (*P* = 0.140), respectively (**Table 2**). The ORRs in patients treated with and without rituximab were 88.3% and 75.0%, respectively. The complete remission rates (CRRs) of the low risk and high risk groups were 93.9% and 81.1% (*P* = 0.044), respectively. The median EFS, PFS, and OS were not reached. The EFS at 3 years for all treated patients was 81.2% (**Figure 1A**). The EFSs at 3 years in the low risk and high risk groups were 95.7% and 71.8%, respectively, [*P* < 0.01, hazard ratio (HR): 0.29, 95% confidence interval (CI): 0.13–0.66, respectively] (**Figure 1B**). The PFS at 3 years for all treated patients was 82.8% (**Figure 2A**). The PFSs at 3 years in the low risk and high risk groups were 95.7% and 74.3%, respectively, (*P* < 0.01, HR: 0.31, 95% CI: 0.13–0.71), respectively (**Figure 2B**). The 3-year PFSs in patients treated with and without rituximab were 82.7% and 82.5%, respectively. The cause of death was Burkitt lymphoma for 2 patients (4.1%) in the low risk group and 7 patients (8.9%) in the high risk group during follow-up. The OS at 5 years for all treated patients was 88.8% (**Figure 3A**). The overall survival at 5 years in the low risk and high risk groups were 93.3% and 85.7% (*P* = 0.129, HR: 0.37, 95% CI: 0.11–1.24), respectively (**Figure 3B**). The patients were followed-up in the outpatient clinic *via* telephone. The median follow-up for all patients was 43.2 (95% CI: 34.2–52.1) months.

**Table 2:** Summary of clinical responses

Outcome	Low risk, *n* (%)	High risk, *n* (%)	*P*
Best overall response, *n* (%)			
Complete remission	46 (93.9%)	60 (81.1%)	0.044*
Partial remission	2 (4.1%)	6 (8.1%)	/
Stable disease	0 (0)	0 (0)	
Progressed disease	1 (2.0%)	8 (10.8%)	
Objective response rate	98.0%	89.2%	0.140
Cycles			
Median (range)	6 (4~8)	6 (4~8)	/
Therapy duration (months)			
Median	3.8	4.3	/
Range	1.6~6.5	2.2~9.4	
Follow-up (months)			
Median	44.3	39.8	/
95% CI	30.1~58.5	30.1~49.5	

*P < 0.05 was considered statistically significant.

The incidences of all AEs and grade III to IV AEs in the low risk and high risk groups are summarized in **Table 3**. All patients were prophylactically treated with granulocyte colony-stimulating factor 24–48 h after the completion of chemotherapy until the absolute neutrophil count was more than 1,000/mL. The principle AEs were hematological and gastrointestinal events, including leukopenia (98.4%), anemia (97.6%), thrombocytopenia (83.7%), fatigue (52.8%), dyspepsia (54.5%), nausea (22.0%), and elevated transaminase levels (48.0%). The major grade 3–4 AEs included leukopenia (91.9%), anemia (58.5%), thrombocytopenia (73.2%), and febrile neutropenia (48.8%). Twenty-four (49.0%) and 52 (70.3%) treatment-related events led to treatment delays or dose reductions in the low risk and high risk groups, respectively. The common events resulting in treatment delays included severe febrile neutropenia requiring empirical antibiotics (47.2%), severe anemia requiring red blood cell transfusion (26.0%), and severe thrombocytopenia requiring platelet transfusion (37.4%). One patient discontinued chemotherapy because of cardiac surgery for frequent ventricular tachycardia. Two patients received changes in treatment due to severe intolerant AEs without progression. We observed 4 cases (3.3%) of septic shock after chemotherapy. In our study, 11 patients died during the follow-up. No treatment-related death occurred in the low risk group, while 2 treatment-related deaths occurred for patients with severe infection in the high risk group. The treatment-related mortality was 1.63%. A summary of dead cases is presented in **Table 4**.

**Table 3:** Summary of adverse events

Events, *n* (%)	Low risk (*n* = 49)		High risk (*n* = 74)		Total (*n* = 123)	
	Any grade	Grade 3~4	Any grade	Grade 3~4	Any grade	Grade 3~4
Any adverse event	49 (100%)	46 (93.8%)	74 (100%)	70 (94.6%)	123 (100%)	116 (94.3%)
Hematological toxic effects						
Anemia	47 (95.9%)	21 (42.9%)	73 (98.6%)	51 (68.9%)	120 (97.6%)	72 (58.5%)
Leukopenia	47 (95.9%)	43 (87.8%)	74 (100%)	70 (94.6%)	121 (98.4%)	113 (91.9%)
Thrombocytopenia	38 (77.5%)	30 (61.2%)	65 (87.8%)	60 (81.1%)	103 (83.7%)	90 (73.2%)
Febrile neutropenia	19 (38.8%)	19 (38.8%)	41 (55.4%)	41 (55.4%)	60 (48.8%)	60 (48.8%)
Non-hematological toxic effects						
Fatigue	22 (44.9%)	0	43 (58.1%)	0	65 (52.8%)	0
Diarrhea	5 (10.2%)	0	6 (8.1%)	1 (1.4%)	11 (8.9%)	1 (0.8%)
Dyspepsia	22 (44.9%)	0	45 (60.8%)	0	67 (54.5%)	0
Nausea	6 (12.2%)	0	21 (28.4%)	1 (1.4%)	27 (22.0%)	1 (0.8%)
Emesis	4 (8.2%)	0	13 (17.6%)	1 (1.4%)	17 (13.8%)	1 (0.8%)
Stomatitis	8 (16.3%)	1 (2%)	9 (12.2%)	1 (1.4%)	17 (13.8%)	2 (1.6%)
Elevated transaminases	27 (55.1%)	1 (2%)	32 (43.2%)	1 (1.4%)	59 (48.0%)	2 (1.6%)
Elevated serum bilirubin	2 (4.1%)	0	9 (12.2%)	0	11 (8.9%)	0
Serum creatinine increased	1 (2%)	0	4 (5.4%)	0	5 (4.1%)	0

**Table 4:** Summary of clinical features in 11 deaths

Characteristics	*n* (%)
Age >60 years	1 (9.1%)
LDH >250 U/L	8 (72.7%)
IPI ≥ 3	7 (63.6%)
Bone marrow involvement	4 (36.4%)
Bowel involvement	5 (45.5%)
EBER	
Negative	4 (36.4%)
Unavailable data	7 (63.6%)
Dose reduction during chemotherapy	2 (18.2%)
Best tumor response in first line CODOX-M therapy	
Complete remission	5 (45.5%)
Progressed disease	6 (55.5%)
Cause of death	
Treatment related death	2 (18.2%)
Died for progressed disease	9 (81.8%)

LDH, lactate dehydrogenase; IPI, international prognosis index; EBER, Epstein-Barr virus-encoded small nuclear early region; CODOX-M, cyclophosphamide, vincristine, doxorubicin, methotrexate.

## Discussion

In this large, single institution retrospective analysis covering 1 decade and 123 patients, we presented preserved efficacy and decreased toxicity with modified R-CODOX-M/IVAC chemotherapy regimens featuring reduced dosages for each course of treatment, but increased cycles of courses. Sporadic Burkitt lymphoma, accounting for 1.2% of adult NHLs, is the most common variant of Burkitt lymphoma in China^12^. Chemotherapy regimens used for adult Burkitt lymphoma patients have evolved from those used in children. The development of several short-term, intensive, multiagent systemic chemotherapies, which are mainly composed of combinations of doxorubicin, alkylators, vincristine, methotrexate, etoposide, and cytarabine, has significantly improved the survival of Burkitt lymphoma patients over the past 2 decades^13^. The 2-year EFS for CODOX-M/IVAC regimens ranged from 59.5% to 92%, and the OS varied from 69.9% to 83.3%^2,3^, with a marked improvement in survival seen during the past decades^14^. Thus, short, intensive, multiagent chemotherapy with CNS prophylaxis is currently the standard treatment for adult patients with Burkitt lymphoma. In China, the latest official guidelines for hematological malignancies recommend R-CODOX-M/IVAC or R-HyperCVAD chemotherapy regimens as prior treatment^15^. However, this first class expert recommendation does not come from clinical trials of Chinese Burkitt lymphoma patients and is mainly based on research in Europe and the United States, countries that have completely adopted the doses and cycles of the original regimens. Though it promoted improvements in survival, the high intensity, short duration treatment strategy was associated with significant neurological toxicity, myelosuppression, and mucositis in almost all patients. Sepsis occurred in 22% of patients, and the majority of patients treated with each regimen required blood product support, especially those high risk patients who received IVAC chemotherapy^3^. Twenty-one percent of patients were unable to complete therapy, and only 43% of high risk patients received full dose therapy^16^.

It was therefore difficult to implement this treatment plan safely in clinical practice. To reduce toxicity, the CODOX-M/IVAC regimen was modified by different clinicians. The LY10 study reduced the methotrexate dose to 3 g/m^2^ and kept the rest the same, which reduced mucositis but still resulted in an 8% (9/110) treatment-related mortality^4^. Lacasce et al.^16^ modified the original Magrath regimens by changing cyclophosphamide from 800 mg/m^2^ on day 1 and 200 mg/m^2^ on days 2–5 to 800 mg/m^2^ on days 1–2, decreasing methotrexate from 6.7 g/m^2^ to 3 g/m^2^, decreasing IT cytarabine from 70 mg to 50 mg, and increasing doxorubicin from 40 mg/m^2^ to 50 mg/m^2 16^. The CRR was 86%, with 2-year PFS and OS values of 64% and 85%, respectively, in this prospective study of 14 adult Burkitt lymphoma patients. In addition to severe myelotoxicity, their modified treatment regimen was associated with no grade 3–4 neuropathy and only 1 episode of grade 3–4 mucositis. The AMC 048 study adopted this modified CODOX-M/IVAC regimen and used it for 34 patients with HIV-positive Burkitt lymphoma treated with rituximab^17^. With a 68% protocol completion, the toxicity profile was similar to that in Lacasce’s study, resulting in a grade 3–4 toxicity of 79% and no grade 3–4 mucositis^17^. Corazzelli et al.^18^ evaluated modified CODOX-M/IVAC regimens mainly containing the addition of rituximab, a decreased dose of methotrexate of 3 g/m^2^, and a vincristine dose of 2 mg with omission on day 15. With 80% of patients finishing the protocol, the modified regimens yielded a higher CRR, EFS, and OS than standard Magrath treatment, but severe toxicity was still universal, with 45% of patients experiencing febrile neutropenia and 53% of patients experiencing gastrointestinal toxicity^18^. Evens et al.^5^ reported similar CRR and PFS values as those obtained from the Corazzelli study, but severe cardiac events occurred due to the high dose of rituximab. The modification of CODOX-M backbone, such as CALGB 9251 and 10002 regimens^9,10^, also presented similar long-term outcomes and treatment related mortalities. The CALGB protocol treated patients with longer course and high intensity. The low intensity EPOCH-R showed that the duration of exposure to anthracyclines above a certain threshold level may be more important than the peak dose^7^. Thus, these prospective studies confirmed the benefit of rituximab in Burkitt lymphoma, but exploration of toxicity management in a broad population is still needed.

We assumed that reduced doses and longer courses of CODOX-M regimen might result in similar outcomes and more tolerant toxicities, with higher treatment compliances. Our modified regimen reduced the doses of cyclophosphamide (500 mg/m^2^ on days 1–2) and methotrexate (2 g/m^2^) used in R-CODOX-M, and reduced the doses of ifosfamide (1.5 g/m^2^ on days 1–3) and cytarabine (2 g/m^2^ q,12 h on the first day) from those used in R-IVAC. In our study, both the 2-year PFS and OS in the low risk and high risk groups (95.7% *vs.* 74.3% for PFS and 97.7% *vs.* 88.3% for OS) were higher than those in the LY06 and LY10 studies^2,4^. It should be noted that the baseline characteristics of patients were different from those of the LY06 and LY10 studies; we had fewer patients with bone marrow involvement (8.9% *vs.* 40%~46%) and fewer patients with CNS involvement (1.6% *vs.* 10%~11%), which might explain the difference. However, patients did not receive rituximab in either the LY06 or LY10 study. In our study, the majority of patients received rituximab, which might have improved the efficacy. In a prospective study incorporating rituximab into the original CODOX-M/IVAC regimen in 2013^5^, the CRR was 92%, and the 2-year PFS and OS for all 25 patients were 80% and 84%, respectively (low risk: both 100%; high risk: 76% and 81%, respectively), despite 32% of patients having bone marrow involvement and 12% of patients having CNS involvement, which was similar to our results. In addition, compared with the results reported in the same period in China, our modified R-CODOX-M/IVAC regimen also showed advantages in efficacy. Yang et al.^19^ retrospectively analyzed 29 Chinese adult Burkitt lymphoma patients treated with Hyper-CVAD, which resulted in a poor prognosis with a 5-year overall survival of 49%. In our study, modification of the doses and cycles was not associated with a less favorable outcome.

It is worth mentioning that several of the most concerned severe treatment-related toxicities were reduced in our study. Grade 3 or 4 mucositis ranged from 7.1% to 48% in modified CODOX-M regimens in a previous study, with a 3 g/m^2^ dose of methotrexate. In our cohort, no serious mucositis occurred because of the reduced dose of high dose methotrexate of 2 g/m^2^. Although the doses of ifosfamide and cytarabine were reduced from those used in the original IVAC regimen, grades 3–4 myelosuppression still occurred in most cases. Almost half of the patients suffering severe febrile neutropenia required empirical antibiotics, but only approximately one-third of the patients needed blood transfusion, and four cases (3.3%) of septic shock occurred after chemotherapy, with all patients completing the planned therapy. The treatment-related mortality (2/74) of high risk patients receiving R-CODOX-M/IVAC chemotherapy was one-third of that seen in the LY06 and LY10 studies (3/40 and 8/76, respectively)^2,4^. Among 11 deceased cases, 6 of them suffered PD, and the major cause of death was tumor progression. However, the blood and tumor samples for molecular and genomic analyses of the deceased cases were not available. In the future, prospective trials should explore the molecular events for severe adverse events and disease progression.

The major limitation of this study was its retrospective nature and its heterogeneity in baseline risk and treatment factors, which may have led to potential bias. Nonetheless, these findings added to the growing body of non-randomized data demonstrating the efficacy and safety profile of modified R-CODOX-M/IVAC regimens in patients. The main strength of the present study was that it is the first report of modified R-CODOX-M/IVAC regimens in the Chinese population, including a large number of real-world adult Burkitt lymphoma patients; thus, these findings might be generalizable to a broad population of patients, although prospective phase III clinical trials are needed for confirmation.

## Conclusions

Our modified R-CODOX-M/IVAC chemotherapy regimen was effective for sporadic HIV-negative Burkitt lymphoma patients in the Chinese population, and had lower toxicity than the standard R-CODOX-M/IVAC regimen, suggesting that it may be more suitable for clinical application and deserves further prospective evaluations.
