# Supplementary material for: Modified R-CODOX-M/IVAC chemotherapy regimens in Chinese patients with untreated sporadic Burkitt lymphoma
Source: Cancer Biol Med. 2021 Aug 15;18(3):833–40. doi: 10.20892/j.issn.2095-3941.2020.0314 (PMC8330526; doi:10.20892/j.issn.2095-3941.2020.0314)
Supplement: Supplementary file 1 [file cbm-18-833-s001.pdf]

Supplementary material

Table S1 The schedule of modified R-CODOX-M/IVAC regimens

Drug	Dosage	Method	Time
CODOX-M			
Cyclophosphamide	500 mg/m^2	IV	Day 1, 2
Vincristine	1.5 mg/m^2 (maximum dose of 2 mg)	IV	Day 1
Doxorubicin	50 mg/m^2	IV	Day 1
Methotrexate	2,000 mg/m^2	IV	Day 3
	Methotrexate 100 mg/m^2 IV loading dose bolus, followed by 1,900 mg/m^2 IV infusion administrated over the next 12 h. Leucovorin rescue began 24 h from the start of methotrexate treatment		
Methotrexate	15 mg	IT	Day 1
Cytarabine	30 mg	IT	Day 1
IVAC			
Ifosfamide	1,500 mg/m^2	IV	Day 1–3
Mesna	200 mg/m2	IV	Q4H, day 1–3 from the start of ifosfamide
Etoposide	100 mg/m^2	IV	Day 1–3
Cytarabine	2,000 mg/m^2	IV	Q12H, day 2
Methotrexate	15 mg	IT	Day 1
Cytarabine	30 mg	IT	Day 1
Biological agents			
Rituximab	375 mg/m^2	IV	Day 1
G-CSF	5 μg/kg/day	SC	24 h after chemotherapy completion

IV, indicates intravenous; IT, intrathecal; SC, subcutaneous; G-CSF, granulocyte colony-stimulating factor. All cycles were repeated at 21-day intervals.
